# Prognostic significance of bone marrow and spleen ^18^F-FDG uptake in patients with colorectal cancer

**DOI:** 10.1038/s41598-021-91608-2

**Published:** 2021-06-09

**Authors:** Jae-Hoon Lee, Hye Sun Lee, Soyoung Kim, Eun Jung Park, Seung Hyuk Baik, Tae Joo Jeon, Kang Young Lee, Young Hoon Ryu, Jeonghyun Kang

**Affiliations:** 1 Department of Nuclear Medicine, Gangnam Severance Hospital, Yonsei University College of Medicine, Seoul, Republic of Korea; 2 Biostatistics Collaboration Unit, Yonsei University College of Medicine, Seoul, Republic of Korea; 3 Department of Surgery, Gangnam Severance Hospital, Yonsei University College of Medicine, 211 Eonju-ro, Gangnam-gu, Seoul, 06273 Republic of Korea; 4 Department of Surgery, Severance Hospital, Yonsei University College of Medicine, Seoul, Republic of Korea

**Keywords:** Cancer imaging, Colon cancer, Rectal cancer

## Abstract

Serum inflammatory markers are used in the prognostication of colorectal cancer (CRC); however, the corresponding role of positron emission tomography (PET)-derived inflammatory markers remains unclear. This study aimed to investigate the prognostic value of ^18^F-fluorodeoxyglucose (FDG) uptake in the bone marrow and spleen of patients with CRC and evaluate the relationship between FDG uptake estimates in these organs and serum inflammatory markers. In total, 411 patients who underwent preoperative FDG PET/computed tomography (CT) within 1 month of surgery were enrolled. The mean standardized uptake values of the bone marrow and spleen were normalized to the value of the liver, thereby generating bone marrow-to-liver uptake ratio (BLR) and spleen-to-liver uptake ratio (SLR) estimates. The value of BLR and SLR in predicting overall survival (OS) was assessed using the Cox proportional hazards model. The correlation between BLR or SLR and neutrophil-to-lymphocyte ratio (NLR) was evaluated. The predictive accuracy of BLR alone and in combination with SLR was compared using the integrated area under the receiver operating characteristic curves (iAUC). In the univariate analysis, BLR (> 1.06) and SLR (> 0.93) were significant predictors of OS. In the multivariate analysis, BLR was an independent predictor of OS (hazard ratio = 5.279; *p* < 0.001). Both BLR and SLR were correlated with NLR (*p* < 0.001). A combination of BLR and SLR was better than BLR alone at CRC prognostication (iAUC, 0.561 vs. 0.542). FDG uptake estimates in the bone marrow and spleen may be useful imaging-derived biomarkers of systemic inflammation, supporting CRC prognostication.

## Introduction

Colorectal cancer (CRC) is among the most common causes of death worldwide^1^. Although incidence and mortality rates remain high, survival outcomes of patients with CRC have improved owing to advances in surgical techniques, advent of improved chemotherapeutic drugs, and refinements in radiation therapy^2^. Reliable prognostic biomarkers and accurate pretreatment evaluation tools are also paramount to the management of CRC.

^18^F-fluorodeoxyglucose (FDG) positron emission tomography/computed tomography (PET/CT) is widely used in CRC staging, treatment response assessment, recurrence diagnosis, and prognostication^3^. PET/CT is mostly used for primary lesion assessments; however, it can also be used to investigate non-oncologic pathologies such as infection and inflammation^4^. Recent studies have implicated inflammation as a critical part of tumor microenvironment in various types of cancer, where it affects tumor development, survival, and response to treatment^5^. Consequently, systemic inflammatory surrogate markers such as C-reactive protein levels, neutrophil-to-lymphocyte ratio (NLR), and platelet-to-lymphocyte ratio have been investigated as candidate predictors of CRC prognosis^3,6–8^. Meanwhile, clinical studies have reported a positive correlation between FDG uptake in the bone marrow (BM) and spleen on PET/CT and the levels of serum inflammatory and immune markers^9–11^, suggesting these parameters may be useful in evaluating inflammation and CRC prognostication.

However, the relevance of PET-derived inflammatory markers (i.e., FDG uptake in the BM and spleen) remains unclear in patients with CRC; to date, only two studies have reported regarding the prognostic value of FDG uptake in the BM and spleen^12,13^. Thus, this study aimed to investigate the prognostic value of FDG uptake in the BM and spleen in patients with CRC, along with other clinical and pathologic factors, and evaluate the relationship between FDG uptake estimates in these organs and serum inflammatory markers.

## Methods

Patients who underwent preoperative PET/CT examinations, followed by curative surgical resection of stage I–IV CRC between November 2007 and November 2013 were enrolled in this study. Patients were eligible for the present study if they met the following criteria: (1) pathologically confirmed stage I–IV CRC; (2) availability of preoperative PET/CT scans; and (3) surgery with curative intent, including resection of distant metastasis. Patients were excluded from the present study if they met the following criteria: (1) baseline PET/CT examinations performed more than 1 month before the surgery; (2) diagnosis of hereditary nonpolyposis CRC, ulcerative colitis, or Crohn’s disease; (3) diagnosis of another acute inflammatory disease, or (4) unmeasurable target organ or tumor uptake (Supplementary Fig. S1 online). This retrospective single-center study included a total of 411 patients.

For preoperative staging assessment, blood cell count and carcinoembryonic antigen (CEA) levels were assessed; colonoscopy, chest and abdominopelvic CT, and PET/CT were performed for all enrolled patients. The American Society of Anesthesiology (ASA) grade and body mass index (BMI) were recorded. NLR was based on the white blood cell count. The median interval between baseline PET/CT and surgical resection was 6 days (interquartile range [IQR] 3–10 days). After a preoperative staging workup, all patients underwent curative surgery, including resection of distant metastasis. The T and N histopathologic stages were determined according to the 7th American Joint Committee on Cancer (AJCC) staging guidelines. The presence of lymphovascular invasion (LVI), microsatellite instability (MSI), and KRAS mutations was evaluated using surgical specimens. Methodological details of MSI^14^ and KRAS^15^ mutation evaluation have been described elsewhere. After curative surgery, adjuvant chemotherapy was performed, as suitable, and all patients underwent routine follow-up assessments, including blood tests and conventional chest and abdominopelvic CT, every 3–6 months.

The study protocol adhered to the ethical standards of the institutional and/or national research committees and 1964 Helsinki Declaration and its later amendments. The institutional review board of the Gangnam Severance Hospital, Yonsei University College of Medicine, approved this study and waived the requirement for written informed consent owing to the retrospective study design.

### Image acquisition and processing

All patients were asked to fast for at least 6 h before undergoing PET/CT, which was performed when blood glucose levels were < 150 mg/dL. Sixty minutes after intravenously administering FDG (5.5 MBq/kg of body weight), PET/CT was performed using a hybrid PET/CT scanner (Biograph 40TruePoint or Biograph mCT 64, Siemens Healthcare Solutions USA, Inc., Knoxville, TN). Low-dose CT data were first obtained for attenuation correction using the automatic dose modulation with a reference of 40 mA and 120 kV without contrast enhancement. The PET data were then acquired from the skull base to the proximal thigh for 3 min per bed position in a three-dimensional mode. The PET images were reconstructed using the ordered subset expectation maximization with attenuation that involved two iterations and 21 subsets. The matrix size and thickness of the reconstructed PET images were 128 × 128 mm and 5 mm, respectively.

### Image analysis

PET/CT images were retrospectively assessed by two board-certified nuclear medicine physicians who were blinded to patient outcomes. Discrepancies in assessments between the physicians were resolved by consensus. First, a volume of interest (VOI) was manually drawn around the primary tumor, taking care to avoid physiologic FDG uptake, especially in the urinary bladder and both urinary tracts. The maximum standardized uptake value (SUV_max_) of the primary tumor was calculated as follows: (decay-corrected activity per milliliter of tissue volume)/(injected FDG activity per gram of body mass). Subsequently, FDG uptake values in the BM, spleen, and liver were measured. For the BM, the spheroid VOIs were drawn over at least five vertebral bodies of the lumbar and lower thoracic spines. Vertebrae with severe compression, osteoporosis, or postoperative change were excluded from the measurements. For each VOI, an isocontour set at 75% of SUV_max_ was automatically generated and the mean value of the voxels within the isocontour was measured and defined as the BM SUV. For the spleen SUV, one spherical VOI of size 1.5 cm in diameter was placed at the center of the spleen, and the mean SUV was measured. Finally, for liver measurements, a spherical VOI of 2 cm was placed in the right lobe of the normal liver, and the mean SUV was then measured. The tumor-to-liver uptake ratio (TLR), BM-to-liver uptake ratio (BLR), and spleen-to-liver uptake ratio (SLR) were then calculated by dividing the SUV_max_ of the primary tumor, BM SUV, and spleen SUV each by the mean SUV of the liver.

### Statistical analysis

Patients’ clinicopathologic characteristics were examined using a variance test, where appropriate. The chi-square and Fisher exact tests were used to compare categorical variables, as suitable. The Student’s *t* test and Mann–Whitney *U* test were used to compare continuous variables, as suitable.

The primary outcome was overall survival (OS), defined as the time between the day of imaging acquisition and death owing to any cause or the last follow-up date. The Cox proportional hazards regression model was used to assess the association between variables of interest and OS in the univariate and multivariate analyses. Variables with *p* values < 0.05 in the univariate analysis were included in the multivariate analyses using backward stepwise selection. Patients were dichotomized into “high” and “low” groups based on BLR and SLR estimates using the optimal cut-off values determined by the largest Χ^2^ in the Mantel–Cox test^16^. OS curves were compared using the Kaplan–Meier method; differences in OS estimates were compared between groups using the log-rank test.

To explore the clinical utility of BLR and SLR as a combined variable, patients were divided into four groups: low BLR and low SLR (group 1), low BLR and high SLR (group 2), high BLR and low SLR (group 3), and high BLR and high SLR (group 4). Differences in the levels of serum inflammatory markers and OS estimates were evaluated among these four groups. Moreover, the accuracy of PET-derived biomarkers in predicting OS was compared between BLR alone and in combination with SLR using an integrated time-dependent area under the curve (iAUC). Differences in iAUC estimates between the PET-derived parameters were measured using bootstrapping with a resampling number of 1,000.

Continuous variables are reported as medians with an IQR, unless otherwise specified. Spearman’s correlation coefficients were calculated to assess the correlation between two variables. The Mann–Whitney U test and Kruskal–Wallis test were used to compare median values among groups. All statistical analyses were conducted using GraphPad Prism 8 (GraphPad Software, La Jolla, CA, USA). Statistical significance was set at *p* values < 0.05.

## Results

A total of 411 (247 men) patients with CRC were included in this study (Table 1). On preoperative FDG PET/CT, the primary colon cancer demonstrated higher FDG uptake than normal liver, with the median TLR of 4.65. In contrast, the BM and spleen demonstrated lower overall FDG uptake than the liver, and the median BLR and SLR were 0.83 and 0.82, respectively. The median follow-up period was 91.4 (range 2.1–142.6) months. A total of 120 (29.2%) patients died during the follow-up period.

**Table 1:** Patients’ characteristics.

	N (%)
**Sex**	
Female	164 (39.9)
Male	247 (60.1)
**Age (years)**	
< 70	262 (63.7)
≥ 70	149 (36.3)
**ASA**	
1	198 (48.2)
2	159 (38.7)
3 and 4	49 (11.9)
No data	5 (1.2)
**BMI (kg/m**^**2**^**)**	
< 25	305 (74.2)
≥ 25	106 (25.8)
**Preop-CEA (ng/mL)**	
< 5	273 (66.4)
≥ 5	138 (33.6)
**Tumor location**	
Rt. colon	122 (29.4)
Lt. colon	178 (43.3)
Rectum	112 (27.3)
**Tumor size (cm)**	
< 5	218 (53)
≥ 5	193 (47)
**Histologic grade**	
G1	44 (10.7)
G2	324 (78.8)
G3	20 (4.9)
Etc	23 (5.6)
**LVI**	
Absent	292 (71)
Present	108 (26.3)
No data	11 (2.7)
**Number of retrieved LNs**	
< 12	63 (15.3)
≥ 12	348 (84.7)
**Stage**	
I and II	192 (46.7)
III	169 (41.1)
IV	50 (12.2)
**MSI**	
MSS/MSI-low	249 (60.6)
MSI-high	29 (7.1)
No data	133 (32.4)
**KRAS**	
Wild	123 (29.9)
Mutant	59 (14.4)
No data	229 (55.7)
**Chemotherapy**	
No	154 (37.5)
Yes	257 (62.5)
**NLR**	
< 3	264 (64.2)
≥ 3	147 (35.8)
**TLR**	
Median (IQR)	4.65 (3.449–6.475)
**BLR**	
Median (IQR)	0.83 (0.730–0.955)
**SLR**	
Median (IQR)	0.82 (0.740–0.910)

*ASA* American Society of Anesthesiology, *BMI* body mass index, *CEA* carcinoembryonic antigen, *LVI* lymphovascular invasion, *LN* lymph node, *MSI* microsatellite instability, *MSS* microsatellite Stable, *NLR* neutrophil-to-lymphocyte ratio, *TLR* tumor-to-liver ratio, *BLR* bone marrow-to-liver ratio, *SLR* spleen-to-liver ratio, *IQR* Interquartile range.

### Predictors of OS

The prognostic relevance of sex, age, ASA grade, BMI, preoperative CEA level, tumor location, tumor size, histologic grade, LVI, number of retrieved lymph nodes (LNs), AJCC stage, MSI, KRAS mutation status, NLR, TLR, BLR, and SNR was assessed in the survival analysis. The optimal cut-off values for BLR and SLR were determined based on the X-tile program, which yielded 1.06 for BLR and 0.93 for SLR (Supplementary Fig. S2 online). Otherwise, empirical cut-off values were used for the other continuous variables.

**Figure 1:**
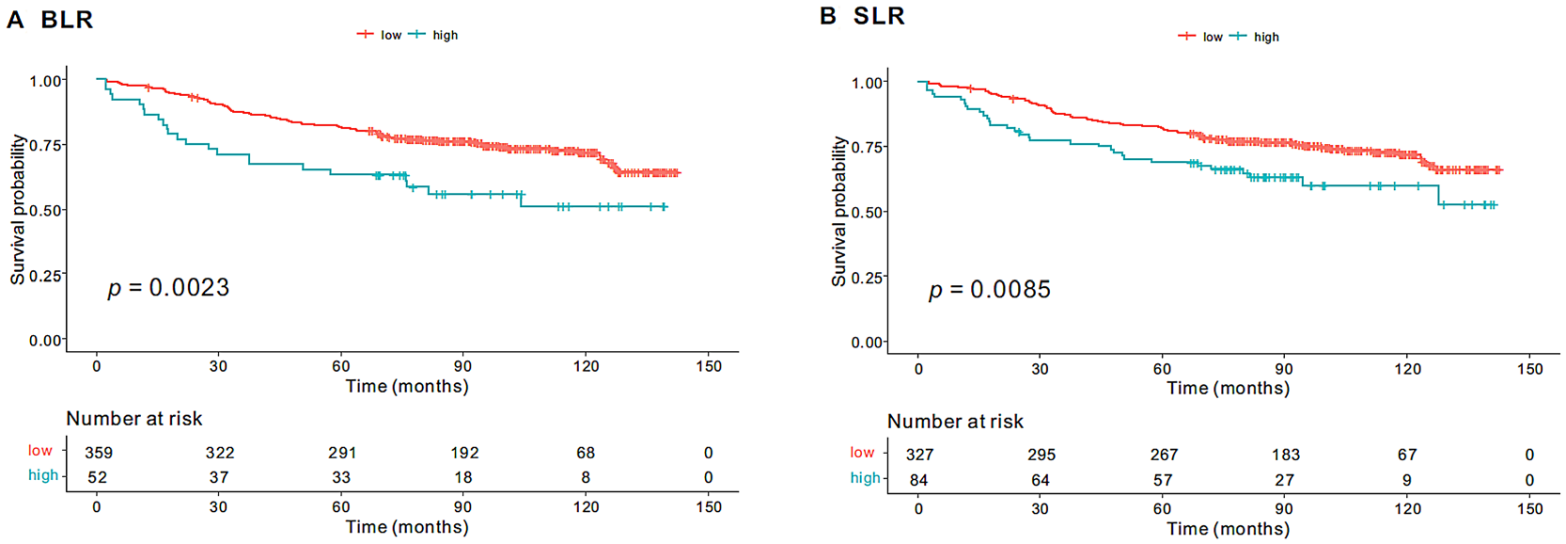
Kaplan–Meier curves of overall survival stratified, according to BLR (**A**) and SLR (**B**).

In the univariate analyses, age (*p* < 0.001), ASA grade (*p* = 0.002), BMI (*p* = 0.001), CEA (*p* = 0.001), tumor size (*p* = 0.023), LVI (absent vs. present, *p* < 0.001), number of retrieved LNs (*p* = 0.031), AJCC stage (I and II vs. IV, *p* < 0.001), postoperative chemotherapy (*p* < 0.001), BLR (*p* < 0.001), and SLR (*p* = 0.030) were significantly associated with OS (Table 2). TLR was not associated with OS (*p* = 0.965). The Kaplan–Meier method revealed that patients with higher BLR (> 1.06) had shorter OS than those with lower BLR (89.6 vs. 114.8 months, *p* = 0.002; Fig. 1A). Similarly, patients with higher SLR (> 0.93) had shorter OS than those with lower SLR (98.4 vs. 115.3 months, *p* = 0.008; Fig. 1B).

**Table 2:** Univariate and multivariate analyses associated with the overall survival (n = 411).

	Univariate analysis		Multivariate analysis	
	HR (95% CI)	*p*	HR (95% CI)	*p*
**Sex**				
Female	Ref			
Male	1.12 (0.772–1.625)	0.55		
**Age (years)**				
< 70	Ref		Ref	
≥ 70	2.261 (1.579–3.238)	< 0.001	2.623 (1.772–3.882)	< 0.001
**ASA**				
1	Ref		Ref	
2	1.793 (1.225–2.625)	0.002	1.921 (1.280–2.883)	0.001
3 and 4	1.153 (0.597–2.227)	0.671	0.707 (0.352–1.417)	0.328
No data	1.145 (0.157–8.315)	0.893	0.974 (0.126–7.495)	0.980
**BMI (kg/m**^**2**^**)**				
< 25	Ref		Ref	
≥ 25	0.424 (0.253–0.708)	0.001	0.431 (0.252–0.738)	0.002
**Preop-CEA (ng/mL)**				
< 5	Ref		Ref	
≥ 5	1.793 (1.251–2.57)	0.001	1.339 (0.910–1.972)	0.138
**Tumor location**				
Rt. colon	Ref			
Lt. colon	0.927 (0.605–1.421)	0.730		
Rectum	0.916 (0.571–1.471)	0.719		
**Tumor size (cm)**				
< 5	Ref			
≥ 5	1.516 (1.058–2.172)	0.023		
**Histologic grade**				
G1	Ref			
G2	1.514 (0.788–2.907)	0.213		
G3	1.711 (0.621–4.713)	0.299		
Etc	1.485 (0.564–3.905)	0.423		
**LVI**				
Absent	Ref		Ref	
Present	2.078 (1.429–3.02)	< 0.001	1.602 (1.054–2.435)	0.027
No data	1.621 (0.590–4.45)	0.348	1.485 (0.514–4.286)	0.464
**Number of retrieved LNs**				
< 12	Ref		Ref	
≥ 12	0.628 (0.411–0.960)	0.031	0.407 (0.259–0.639)	< 0.001
**Stage**				
I and II	Ref		Ref	
III	1.501 (0.983–2.291)	0.059	2.056 (1.286–3.287)	0.002
IV	5.437 (3.401–8.692)	< 0.001	5.560 (3.216–9.610)	< 0.001
**MSI**				
MSS/MSI-low	Ref			
MSI-high	0.643 (0.280–1.479)	0.300		
No data	0.962 (0.654–1.415)	0.846		
**KRAS**				
Wild	Ref			
Mutant	1.075 (0.565–2.041)	0.826		
No data	1.336 (0.861–2.073)	0.196		
**Chemotherapy**				
No	Ref		Ref	
Yes	0.523 (0.365–0.750)	< 0.001	0.536 (0.357–0.805)	0.002
**NLR**				
< 3	Ref			
≥ 3	1.432 (0.997–2.057)	0.051		
**TLR**				
Continuous	1.002 (0.934–1.073)	0.965		
**BLR**				
Continuous	4.638 (1.958–10.99)	< 0.001	5.279 (2.337–11.922)	< 0.001
**SLR**				
Continuous	2.686 (1.097–6.574)	0.030		

*HR* hazard ratio, *CI* confidence interval, *ASA* American Society of Anesthesiology, *BMI* body mass index, *CEA* carcinoembryonic antigen, *LVI* lymphovascular invasion, *LN* lymph node, *MSI* microsatellite instability, *MSS* microsatellite Stable, *NLR* neutrophil-to-lymphocyte ratio, *TLR* tumor-to-liver ratio, *BLR* bone marrow-to-liver ratio, *SLR* spleen-to-liver ratio.

Among the variables that were significantly associated with OS in the univariate analyses, age, ASA grade, BMI, CEA, LVI, number of retrieved LNs, AJCC stage, chemotherapy, BLR, and SLR were included in the multivariate analysis (Table 2). The multivariate analysis revealed that age (*p* < 0.001), ASA grade (1 vs. 2, *p* < 0.001), BMI (*p* = 0.002), LVI (absent vs. present, *p* = 0.027), number of retrieved LNs (*p* < 0.001), AJCC stage (I and II vs. III, *p* = 0.002; I and II vs. IV, *p* < 0.001), chemotherapy (*p* = 0.002), and BLR (*p* < 0.001) were independent predictors of OS (Table 2). SLR was not an independent predictor of OS in the multivariate analysis.

### Correlation of BLR and SLR with NLR

BLR and SLR correlated positively with NLR (*r* = 0.27, *p* < 0.001 for BLR; Supplementary Fig. S3A online; *r* = 0.17, *p* < 0.001 for SLR, Supplementary Fig. S3B online). However, TLR was not correlated with NLR (r = 0.08, *p* = 0.088). The high BLR group demonstrated higher NLR than the low BLR group; the median LNRs were 3.16 (IQR, 2.22) and 2.39 (2.22) for the high BLR and low BLR groups, respectively (*p* < 0.001) (Fig. 4A). The high SLR group had higher NLR than the low SLR group; the median LNRs were 2.99 (2.69) and 2.38 (1.52) for the high SLR and low SLR groups respectively (*p* < 0.001) (Fig. 4B).

### Additive prognostic value of BLR and SLR

**Figure 2:**
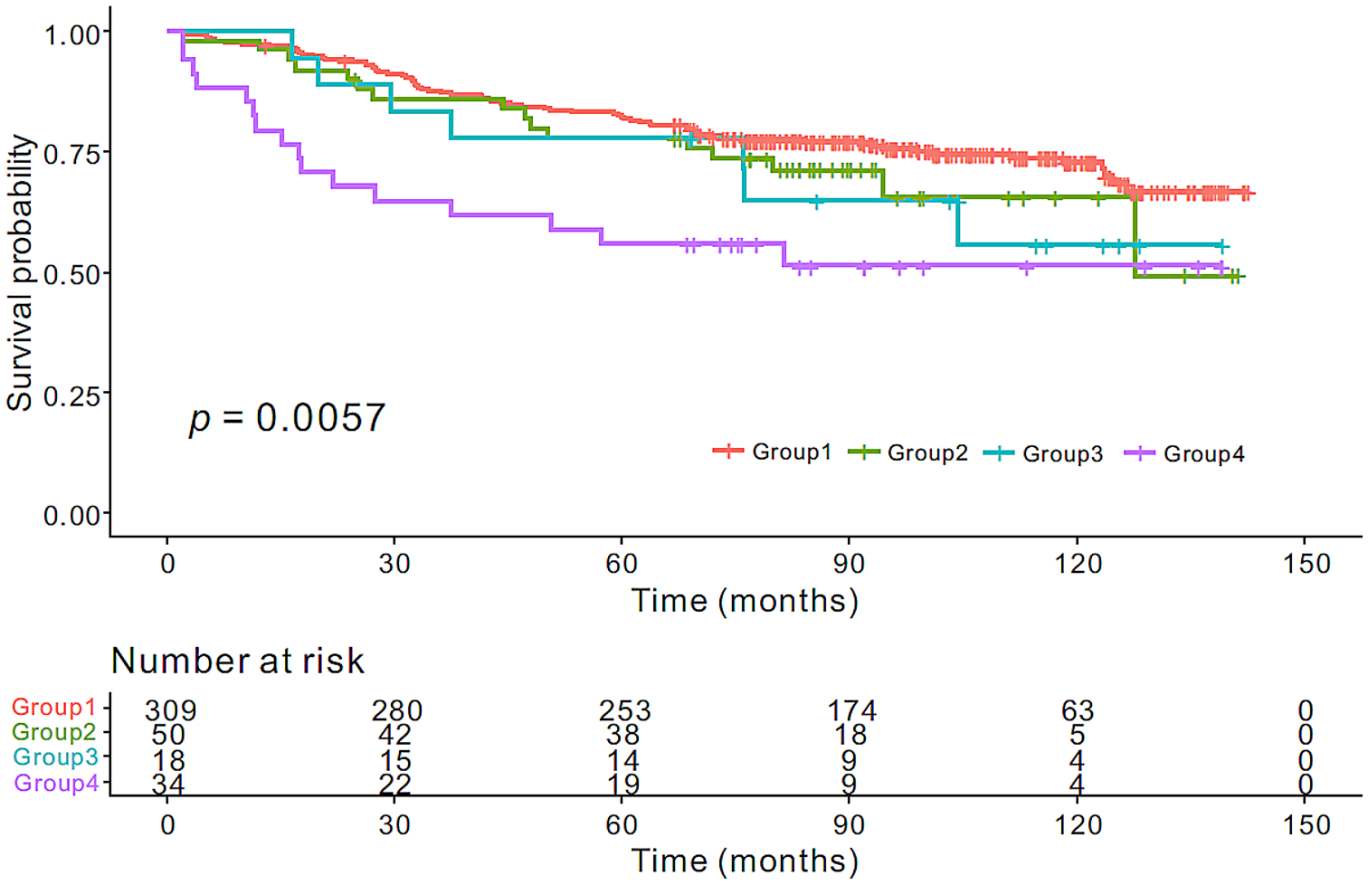
Kaplan–Meier curves of overall survival stratified by the combination of BLR and SLR. Group 1: low BLR and low SLR; Group 2: low BLR and high SLR; Group 3: high BLR and low SLR; Group 4: high BLR and high SLR.

**Figure 3:**
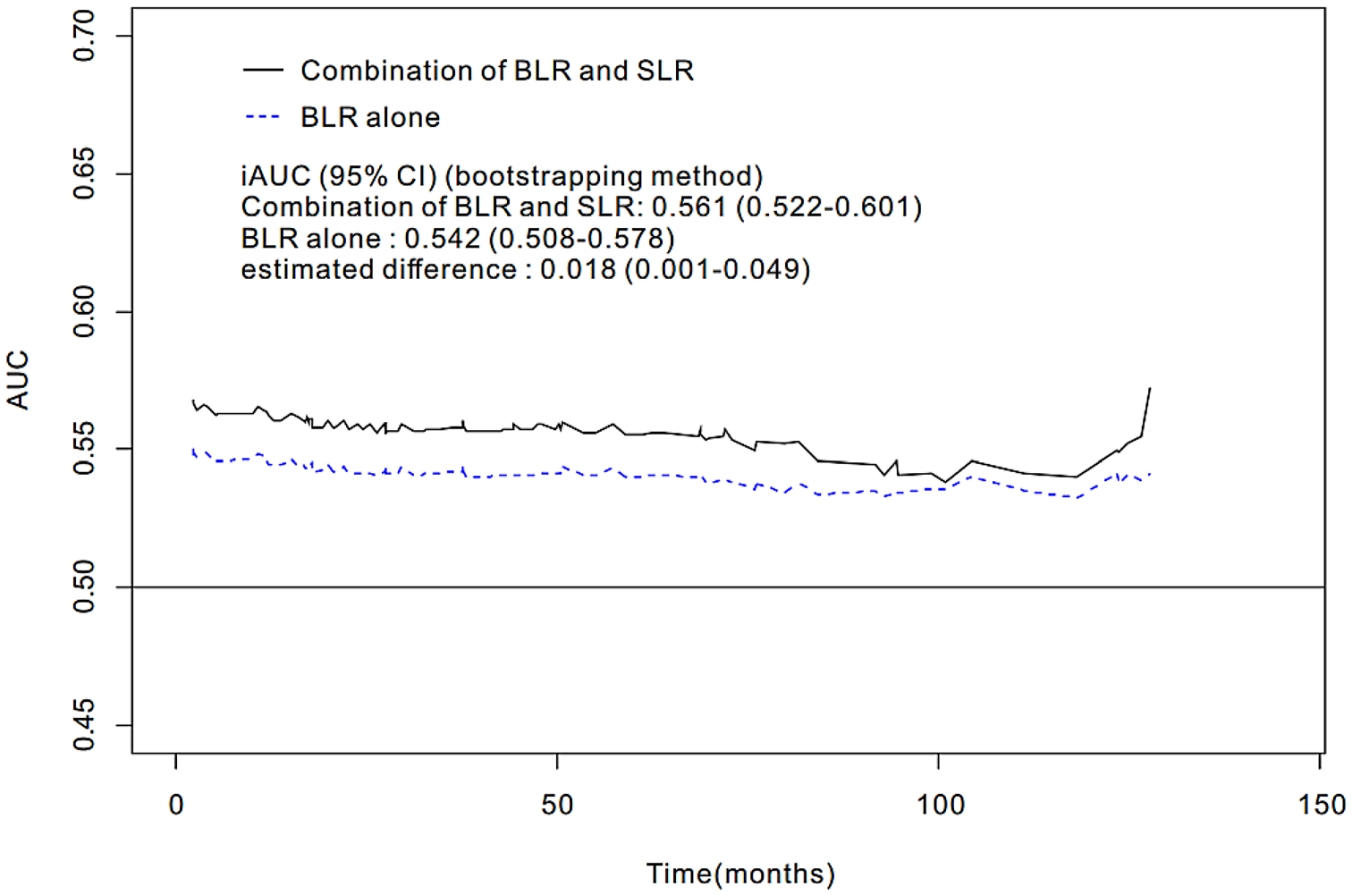
Comparison of integrated AUC (iAUC) between four-group stratification by the combination of BLR and SLR, and between two-group by BLR alone.

**Figure 4:**
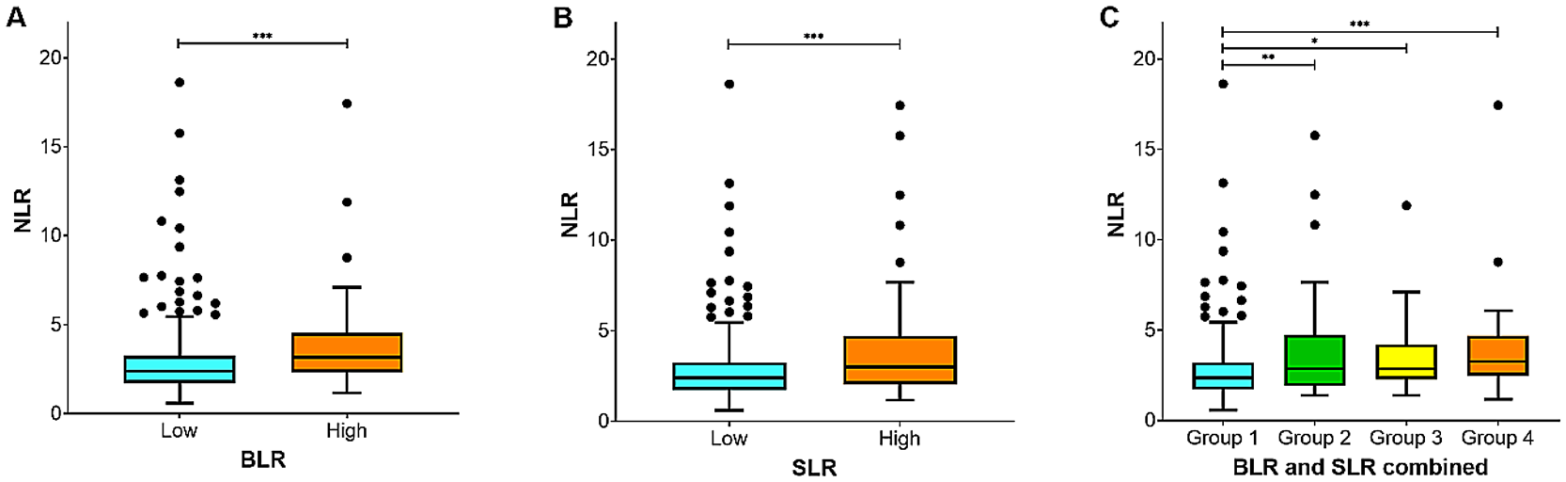
Distribution of NLR according to subgroups stratified by BLR (A), SLR (B), and combination of BLR and SLR (C). *p < 0.05; **p < 0.01; ***p < 0.001.

Patients were divided into low BLR and low SLR (group 1, n = 309), low BLR and high SLR (group 2, n = 50), high BLR and low SLR (group 3, n = 18), and high BLR and high SLR (group 4, n = 34) groups. The univariate analysis demonstrated a significant difference in OS among the four groups (*p* < 0.001). Post-hoc analyses revealed that patients with BLR of > 1.06 and SLR of > 0.93 (group 4) had poorer prognosis than those with BLR of ≤ 1.06 and SLR of ≤ 0.93 (group 1) (*p* < 0.001) (Supplementary Table S1 online). The Kaplan–Meier method revealed poorer OS in group 4 than in group 1 (83.6 vs. 115.9 months; *p* = 0.001) (Fig. 2). In the multivariate analysis, the four-group variable derived from the combination of BLR and SLR was an independent predictor of OS (group 1 vs. group 4, *p* = 0.007) (Supplementary Table S1 online). The iAUC value of the four-group variable (0.562; 95% confidence interval [CI], 0.522–0.601) was superior to that of the two-group variable based on BLR (0.542; CI, 0.508–0.578) throughout the observation period, with an estimated difference of 0.018 (00.001–0.049) (Fig. 3). NLR was significantly different among the four groups; post-hoc analysis revealed that group 1 had significantly lower NLR than the other three groups (Fig. 4C).

## Discussion

The present study showed that both BM and spleen uptake of FDG on preoperative PET/CT may be useful biomarkers of systemic inflammation associated with CRC prognosis. BLR was independently associated with OS in the multivariate analysis. SLR was a significant predictor of OS in the univariate analysis but not in the multivariate analysis. Both BLR and SLR were positively correlated with NLR, which is a systemic inflammatory marker. Moreover, a combination of BLR and SLR was superior to BLR alone in OS prognostication.

BM is vital for hematopoiesis and neutrophil production, which is regulated by the release of the cytokine granulocyte colony-stimulating factor^17^. Previous studies have shown that FDG uptake by the BM is increased in several types of cancer and that it correlates with the levels of serum cytokines, C-reactive protein, and other hematologic parameters, which may be indicative of the BM activation in response to tumor-associated inflammation^9–11^. Inflammatory markers are present in the tumor microenvironment, and tumors frequently develop at the location of chronic inflammation^5^. Inflammation is involved in tumor progression, and baseline systemic inflammation is associated with poor clinical outcomes in cancer patients^18–20^. Consequently, FDG uptake by the BM may be relevant to CRC prognostication; because FDG uptake can be determined using PET, which is a non-invasive imaging modality, this parameter can be easily integrated into clinical practice and research.

The spleen plays an important role in hematopoiesis and immunity^21^; it filters and stores red blood cells, belongs to the mononuclear phagocyte system, and produces lymphocytes. Diffusely increased FDG uptake in the spleen can be observed in several diseases, including inflammatory and oncological conditions^22^. Previous studies have evaluated the prognostic significance of diffuse splenic uptake in some cancers^23–25^, thus demonstrating that FDG uptake in the spleen observed on baseline FDG PET/CT may help predict disease recurrence or disease-specific mortality, particularly when used along with hematologic or inflammatory parameters; the present findings are consistent with those of these previous studies. Similar to FDG uptake in the BM, that in the spleen may indicate an increase in glucose metabolism owing to immune system activation, which may adversely affect patient outcomes.

In the present study, SLR demonstrated a significant positive correlation with NLR; meanwhile, high SLR was associated with poor survival in patients with CRC, although this association was not evident in the multivariate analysis. Nevertheless, the combination of BLR and SLR exhibited enhanced prognostic performance over BLR alone (iAUC, 0.561 vs. 0.542), suggesting that it may help identify patients at high risk of death and those requiring close monitoring or anti-inflammatory therapy. These findings may be associated with the distinct roles of these two organs in hematopoiesis, suggesting that the assessment of FDG uptake in the BM and spleen may help predict patient outcomes. However, in the present sub-group analysis, group 3 (n = 18) and group 4 (n = 34) were both smaller than group 1 (n = 309) and group 2 (n = 50), which may have skewed the presented estimates. Further large studies are required to validate the present findings.

In the present study, FDG uptake by the primary CRC was not associated with OS in the univariate analysis (*p* = 0.965). Some previous studies have demonstrated that SUV_max_ of the primary tumor may affect CRC prognosis^26,27^; other studies have reported that PET/CT parameters are not associated with either disease-free survival or OS^28–30^. These discrepancies in findings may be because of the variability in tumor SUV measurements, confounded by tumor-associated inflammation. A previous study suggested that higher tumor uptake may reflect tumor cell viability and proliferation, contributing to secondary inflammatory reaction^31^. However, the relationship between tumor and inflammation may differ among patients and tumor types.

To date, only one study with 226 patients with CRC investigated the prognostic value of FDG uptake in the BM in predicting recurrence-free survival after curative resection^13^. Data on the prognostic significance of FDG uptake in the spleen of patients with CRC are also limited; only one study with 161 patients with rectal cancer evaluated the role splenic FDG uptake in death prediction after curative resection^12^. These studies demonstrated that high FDG uptake in the BM and spleen was significantly associated with poor survival, which is concordant with the present findings. However, the former study had a short follow-up period (median 32 months), whereas the latter study examined only FDG uptake in the spleen of patients with rectal cancer. The present study involved a larger population of patients with CRC, demonstrating the potential usefulness of both BM and spleen FDG uptake values for reflecting systemic inflammation levels, which may support CRC prognostication.

This study had several limitations. This was a single-center retrospective study that only included patients whose post-surgical pathological assessment findings were available and therefore may involve some selection bias. Thus, the present findings need to be validated in large multicenter studies. Second, different PET/CT scanners were used in this study, which may have resulted in variations in SUV measurements. However, SUV was normalized to that of the liver, instead of using absolute values, to reduce the impact of inter-instrumental variability. Moreover, relative quantification is practical and convenient for researchers and clinicians alike as one can visually evaluate FDG uptake in the tumor and BM relative to that in the liver without using any specialized software or equipment. Therefore, FDG uptake values, presented as the SUV ratio relative to that in the liver, is a reproducible and generalizable parameter. Nevertheless, further studies are required to elucidate the relationship between the increased SUV of the BM and spleen and systemic inflammation related to tumor cells; such studies may involve the measurement of serum cytokine levels and histopathologic analysis of BM aspirates as the tumor-infiltrating immune cells.

In conclusion, BLR and SLR were significantly associated with CRC prognosis in the univariate analysis; meanwhile, BLR was an independent predictor of OS in the multivariate analysis. Both BLR and SLR demonstrated a significant positive correlation with NLR. Moreover, a combination of BLR and SLR was better in predicting OS than BLR alone. Hence, both BLR and SLR examined on preoperative PET/CT can be useful image-derived biomarkers of systemic inflammation, thus supporting CRC prognostication.

## Supplementary information

Supplementary Information.

## Data Availability

The datasets analyzed in this study are not publicly available owing to ethical and privacy restrictions; they are available from the corresponding author on reasonable request.
