# Supplementary material for: Prognostic significance of bone marrow and spleen 18F-FDG uptake in patients with colorectal cancer
Source: Sci Rep. 2021 Jun 9;11:12137. doi: 10.1038/s41598-021-91608-2 (PMC8190120; doi:10.1038/s41598-021-91608-2)
Supplement: Supplementary file 1 — Supplementary Information. [file 41598_2021_91608_MOESM1_ESM.docx]

**Supplementary information**

**Prognostic significance of bone marrow and spleen ^18^F-FDG uptake in patients with colorectal cancer**

Jae-Hoon Lee^1^; Hye Sun Lee^2^; Soyoung Kim^1^; Eun Jung Park^3^; Seung Hyuk Baik^3^;

Tae Joo Jeon^1^; Kang Young Lee^4^; Young Hoon Ryu^1^; and Jeonghyun Kang*^3^

^1^Department of Nuclear Medicine, Gangnam Severance Hospital, Yonsei University College of Medicine, Seoul, Republic of Korea; ^2^Biostatistics Collaboration Unit, Yonsei University College of Medicine, Seoul, Republic of Korea; ^3^Department of Surgery, Gangnam Severance Hospital, Yonsei University College of Medicine, Seoul, Republic of Korea; ^4^Department of Surgery, Severance Hospital, Yonsei University College of Medicine, Seoul, Republic of Korea


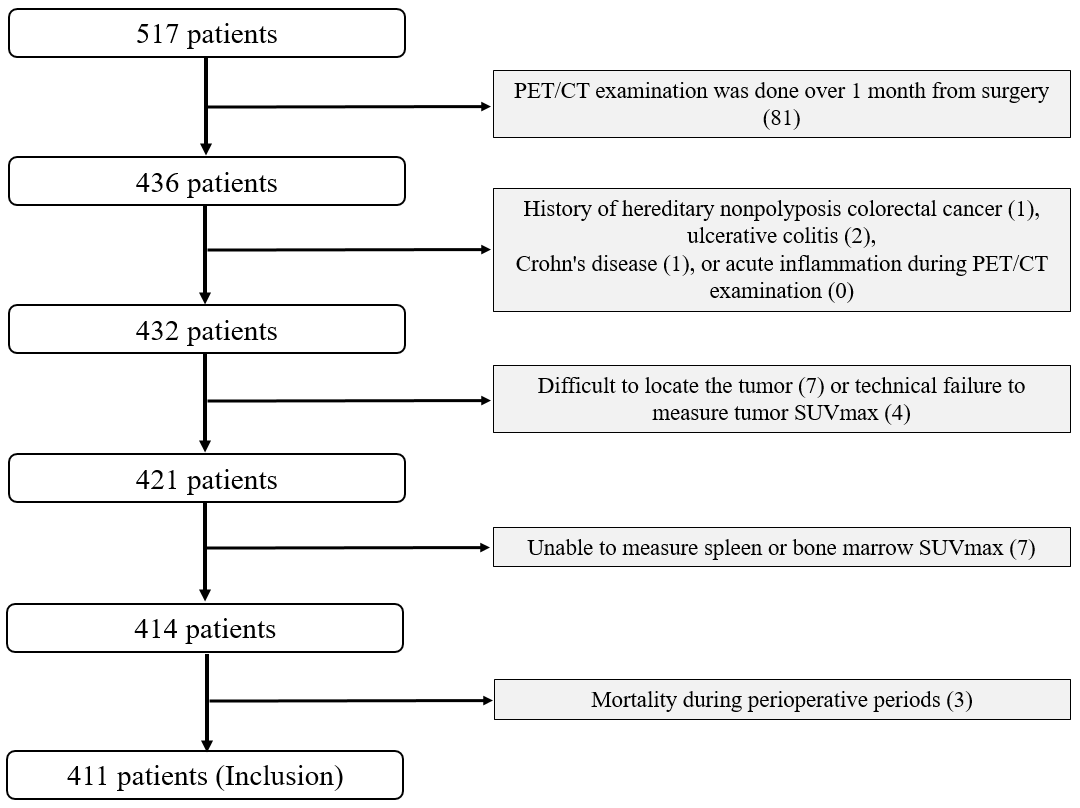


**Supplementary Figure S1.** Patient inclusion criteria used in this study

| **(A) Cut-off value of the BLR** |  |
| --- | --- |
| 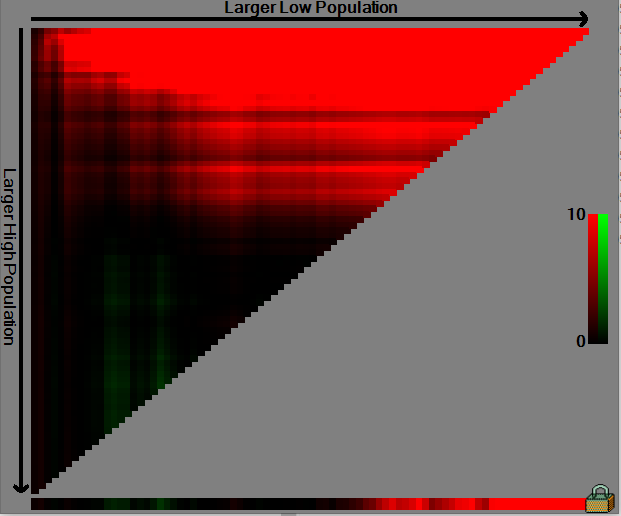 | 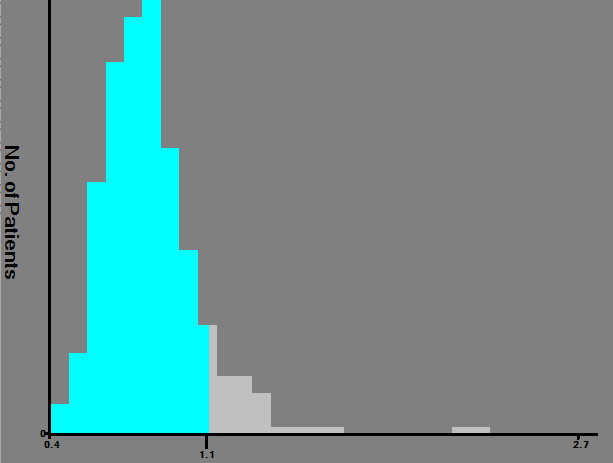 |
| **(B) Cut-off value of the SLR** |  |
| 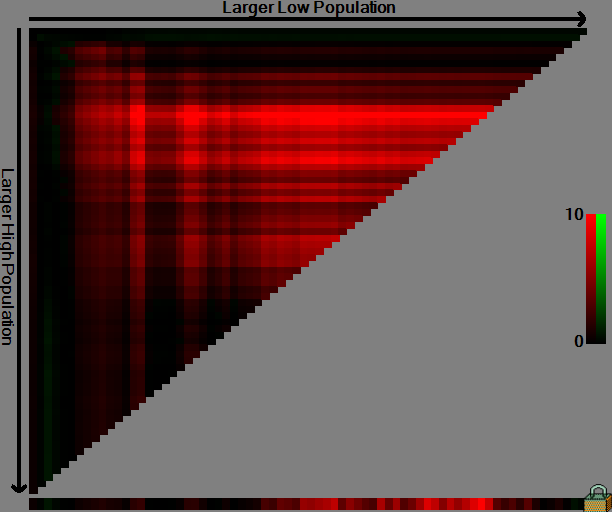 | 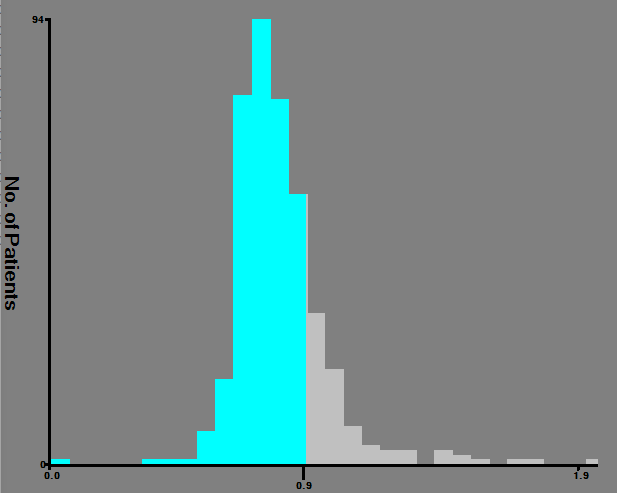 |

**Supplementary Figure S2.** Determining the cut-off values of BLR (A) and SLR (B) using the X-tile program (n=411). X-tile plots of BLR and SLR and the points of the variable coloration of the plot represent the strength of the association at each division ranging from low (dark, black) to high (bright, red, or green). Red represents an inverse association between the expression levels and survival of the variables, whereas green represents a direct association. The optimal cut-off values were defined as the values that produced the largest χ2 in the Mantel-Cox test, and these points were set as 1.06 (BLR) (A) and 0.93 (SLR), respectively. Patients were divided into the two subgroups based on these values on the following survival analysis.


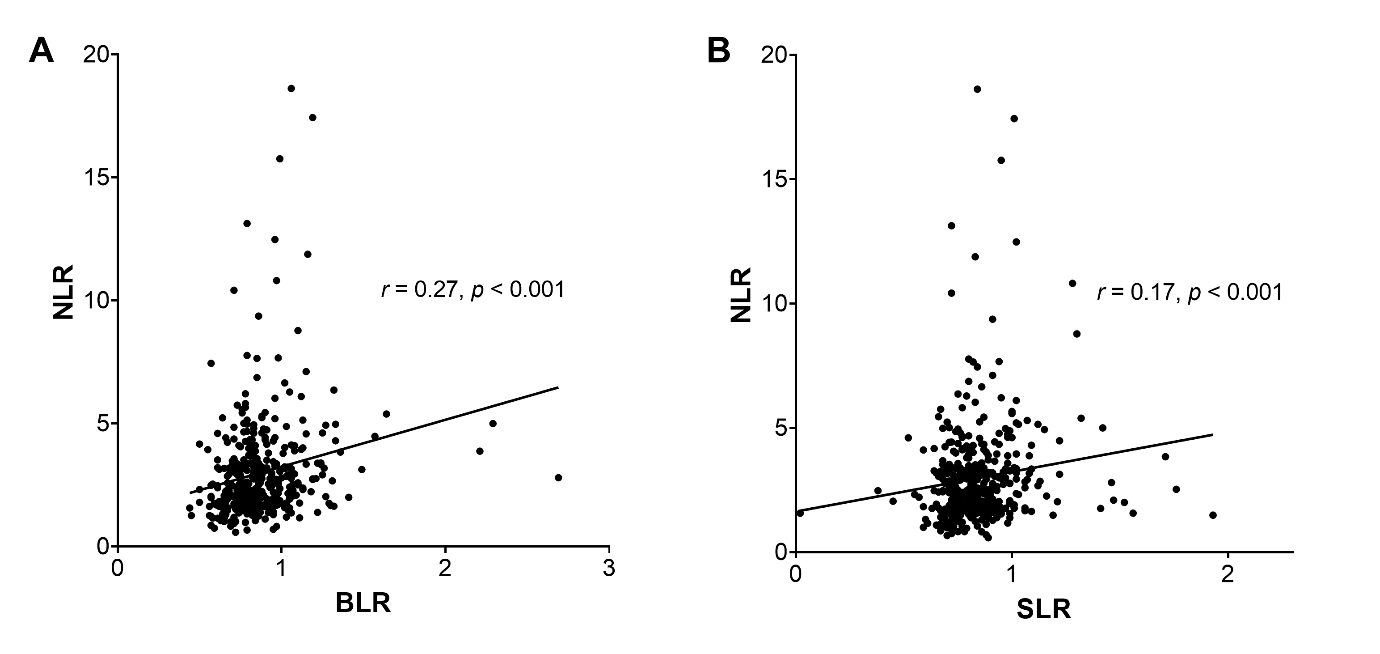


**Supplementary Figure S3.** Correlation between the BLR and neutrophil-to-lymphocyte ratio (NLR) (A) and between the SLR and NLR (B). The correlation was evaluated using Spearman’s correlation coefficient.

**Supplementary Table S1**. Univariate and multivariate analysis associated with the overall survival (n=411)

|  |  | Univariate analysis | | Multivariate analysis | |
| --- | --- | --- | --- | --- | --- |
|  |  | HR (95% CI) | *p* | HR (95% CI) | *p* |
| Age (years) | < 70 | Ref |  | Ref |  |
|  | ≥ 70 | 2.261 (1,579–3,238) | <0.001 | 2.592 (1.732–3.878) | <0.001 |
| ASA | 1 | Ref |  | Ref |  |
|  | 2 | 1.793 (1.225–2,625) | 0.002 | 1.718 (1.143–2.580) | 0.009 |
|  | 3 & 4 | 1.153 (0.597–2.227) | 0.671 | 0.685 (0.338–1.387) | 0.293 |
|  | No data | 1.145 (0.157–8.315) | 0.893 | 0.537 (0.067–4.275) | 0.557 |
| BMI (kg/m^2^) | < 25 | Ref |  | Ref |  |
|  | ≥ 25 | 0.424 (0.253–0.708) | 0.001 | 0.503(0.295–0.857) | 0.011 |
| Preop-CEA (ng/mL) | < 5 | Ref |  |  |  |
|  | ≥ 5 | 1.793 (1.251–2.57) | 0.001 |  |  |
| Tumor size (cm) | < 5 | Ref |  | Ref |  |
|  | ≥ 5 | 1.516 (1.058–2.172) | 0.023 | 1.518 (1.005–2.293) | 0.046 |
| LVI | Absent | Ref |  | Ref |  |
|  | Present | 2.078 (1.429–3.02) | <0.001 | 1.626 (1.061–2.492) | 0.025 |
|  | No data | 1,621 (0.590–4.45) | 0.348 | 1.741 (0.599–5.057) | 0.307 |
| LN numbers | < 12 | Ref |  | Ref |  |
|  | ≥ 12 | 0.628 (0.411–0.960) | 0.031 | 0.379 (0.234–0.613) | <0.001 |
| AJCC Stage | I & II | Ref |  | Ref |  |
|  | III | 1.501 (0.983–2.291) | 0.059 | 2.204 (1.329–3.657) | 0.002 |
|  | IV | 5.437 (3.401–8.692) | <0.001 | 6.699 (3.899–11.511) | <0.001 |
| Chemotherapy | No | Ref |  | Ref |  |
|  | Yes | 0.523 (0.365–0.750) | <0.001 | 0.524 (0.332–0.827) | 0.005 |
| BLR and SLR (n) | Group 1 (309) | Ref |  | Ref |  |
|  | Group 2 (50) | 1.362 (0.795–2.330) | 0.260 | 0.817 (0.448–1.489) | 0.510 |
|  | Group 3 (18) | 1.532 (0.707–3.316) | 0.278 | 0.980 (0.417–2.304) | 0.964 |
|  | Group 4 (34) | 2.494 (1.457–4.268) | <0.001 | 2.151 (1.227–3.770) | 0.007 |

HR=Hazard Ratio, CI=Confidence Interval, SD=Standard Deviation, ASA=American society of anesthesiology; BMI=Body mass index, CEA=Carcinoembryonic antigen, LNI=Lymphovascular invasion; LN=Lymph node.
